# Reactions of two xeric-congeneric species of *Centaurea* (Asteraceae) to soils with different pH values and iron availability

**DOI:** 10.7717/peerj.12417

**Published:** 2021-11-10

**Authors:** Mateusz Wala, Jeremi Kołodziejek, Janusz Mazur, Alicja Cienkowska

**Affiliations:** 1 Department of Geobotany and Plant Ecology, Faculty of Biology and Environmental Protection, University of Lodz, Łódź, Łódź Voivodeship, Poland; 2 Laboratory of Computer and Analytical Techniques, Faculty of Biology and Environmental Protection, University of Lodz, Łódź, Łódź Voivodeship, Poland; 3 Łódź, Łódź Voivodeship, Polska

**Keywords:** *Centaurea*, Iron, Xerothermic grasslands, Congeneric species, Plant nutrition

## Abstract

*Centaurea scabiosa* L. and *C. stoebe* Tausch are known to co-exist naturally in two extremely different types of open dry habitats in the temperate zone, alkaline xerothermic grasslands and acidic dry grasslands. However, knowledge about their preferences to edaphic conditions, including soil acidity (pH), and iron (Fe) availability is scarce. Therefore, experimental comparison of soil requirements (acidic Podzol vs alkaline Rendzina) of these species was carried out. The study was designed as a pot experiment and conducted under field conditions. Fe availability was increased by application of Fe-HBED. Reactions of plants to edaphic conditions were determined using growth measurements, leaf morphometric measurements, chlorosis scoring, chlorophyll content and chlorophyll *a* fluorescence (OJIP) quantification as well as determination of element content (Ca, Mg, Fe, Mn, Zn and Cu). Growth and leaf morphometrical traits of the studied congeneric species were affected similarly by the soil type and differently by the chelate treatment. Increased availability of Fe in Rendzina contrasted the species, as treatment with 25 µmol Fe-HBED kg^−1^ soil promoted growth only in *C. stoebe*. Both species turned out to be resistant to Fe-dependent chlorosis which was also reflected in only minor changes in chlorophyll *a* fluorescence parameters. Both species showed relatively low nutritional demands. Surprisingly, Fe-HBED did not stimulate Fe acquisition in the studied species, nor its translocation along the root:shoot axis. Furthermore, contrary to expectations, *C. scabiosa* took up less Fe from the acidic than alkaline soil. *C. scabiosa* not only absorbed more Ca and Zn but also translocated greater amounts of these elements to shoots than *C. stoebe*. Both species acquired more Mg on Podzol than on Rendzina which suggests adaptation allowing avoidance of aluminum (Al) toxicity on acidic soils. Overall, it seems that *C. scabiosa* prefers alkaline soils, whilst *C. stoebe* prefers acidic ones.

## Introduction

The genus *Centaurea* L. belongs to the dicotyledonous Asteraceae family and is believed to be among the largest and the most taxonomically-challenging genera among Asteraceae (Greuter et al., 2001; Aksoy et al., 2016). Up to date it is known that *Centaurea* includes several hundreds of species (c.a. 400–700; Greuter et al., 2001), divided into three subgenera, *Centaurea sensu stricto*, *Lopholoma* (Cass.) Dobrocz. and *Cyanus* (Mill.) Hayek (Hilpold et al., 2014). All species within *Centaurea* are annual to perennial herbs (only rarely dwarf shrubs; Dostál, 1976), distributed naturally in Eurasia. Species of *Centaurea* occupy widely different open habitats varying in soil type, from inland sand dunes to meadows and from acidic to calcareous grasslands. Habitat preferences for vegetative growth of well-recognized species of *Centaurea* were widely described in the past (Ellenberg, 1991). Some species of *Centaurea* (*e.g*., *C. diffusa* Lam., *C. jacea* L. and *C. stoebe* Tausch) were introduced to non-native areas (*e.g*., North America), where they became weed and/or invasive species (DiTomaso, 2000; Hahn, Buckley & Müller-Schärer, 2012).

Grasslands are non-woody plant communities where edaphic characteristics play crucial role in formation and maintenance of their structure and floristic composition (Puerto & Rico, 1997; Michaud et al., 2012). The mosaic structure of soils and diversified requirements of plant species contribute to species richness of grasslands (Leuchner & Ellenberg, 2017). Various types of grasslands (*e.g*., acidophilous and basiphilous grasslands; Bothe, 2015) establish on different, sometimes extremely contrasting types of soils. Considering chemical characteristics, soil acidity (pH) is among the major factors shaping plant establishment and survival in natural and man-made habitats (Haling et al., 2011; Borhannuddin Bhuyan et al., 2019). Soils with extremely different pH (remarkably acidic or alkaline) differ in other physical-chemical traits, including, among others buffering capacity, water holding capacity and availability of elements (Jentsch & Beyschlag, 2003; Bothe, 2015; Kabała, 2018), including crucial nutrients (Leuchner & Ellenberg, 2017; Lambers & Oliveira, 2019; Schulze et al., 2019). Soil acidification increases availability of cations (aluminum–Al, copper–Cu, iron–Fe, manganese–Mn and zinc–Zn, but not magnesium–Mg and calcium–Ca) due to desorption of elements from soil particles and their dissolution from minerals (Lambers & Oliveira, 2019). Thus, acidic soils provide sufficient amounts of some elements (most notably Fe and Mn) for plants, or even their availability may cause ion-specific toxicity (*e.g*., Al; Strawn, Bohn & O’Connor, 2020). *Vice versa*, plants from alkaline soils encounter shortage of Fe and Mn and do not suffer from Al toxicity, as their solubility is controlled mainly by soil pH (Bothe, 2015). Moreover, alkaline soils are remarkably rich in freely available Ca, whilst sandy acidic soils are poor in this element unless influenced by human activity and marine or alluvial depositions. Availability of Zn and Cu can also be a limiting factor on both acidic and alkaline soils (Lambers & Oliveira, 2019). Additionally, concentration and speciation of nitrogen (NO_3_^–^/NH_4_^+^) and phosphorus (HPO_4_^2–^/H_2_PO_4_^–^) strongly differs alkaline soils from acidic ones (Leuchner & Ellenberg, 2017).

Plant nutritional status is controlled by multilayer mechanisms acting simultaneously in roots and in shoots (Baxter et al., 2008). Although some cations (Ca, Mg and K) are provided to plant cells mainly due to mass flow and diffusion, Fe, Mn, Zn and Cu are acquired by divalent cation transporters with a broad substrate ranges (Korshunova et al., 1999; Lambers & Oliveira, 2019). It implies that at least some micronutrients may compete for transporters and their acquisition by plants depends on local soil elemental ratio. Such mode of action was previously observed in in the case of increased availability of Fe (*e.g*., due to application of chelates, including Fe-HBED) which is known to alter acquisition patterns of Mn and Zn and their allocation on a root-shoot axis (Baxter et al., 2008; Wala et al., 2020). As plant ionome (from cell to whole organism scale) is a complex networked structure (Merchant, 2010), its homeostasis can be disturbed by each element and reestablished by plant response if tolerance buffer is not exceeded. However, pH-dependent ion-specific limitations and co-limitations for plant growth are not well understood yet, especially in the case of habitat-specialized wild-living plants.

Iron (Fe) is a relatively common element and can be found in considerable amounts in various types of soils (Colombo et al., 2014). However, numerous biogeochemical processes lead to formation of Fe fractions that are poorly available for plants (Colombo et al., 2013). Such a situation prompted evolution of Fe acquisition mechanisms in terrestrial plants. Dicotyledonous plant species (including species of *Centaurea*) utilize two-step mechanism based on (1) acidification of rhizosphere by H^+^ extrusion and (2) enzymatic reduction of Fe^3+^ to Fe^2+^ and uptake of the reduced Fe form (Strategy I mechanism; Jeong & Connolly, 2009; Krohling et al., 2016). Additionally, some Strategy I species secrete secondary metabolites (phytosiderophores) chelating Fe (Sisó-Terraza et al., 2016), which greatly helps plants occurring on alkaline soils (due to high soil buffering capacity halting acidification-dependent solubilization of Fe-containing compounds; Grillet & Schmidt, 2017). Disturbances in Fe scavenging process lead to nutritional deficiencies and, in severe cases, to development of chlorosis (Celletti et al., 2016). This, in turn, strongly limits plant primary functioning (*e.g*., photosynthesis and acquisition of nutrients; Venturas et al., 2014; Luna et al., 2018). As soil acidity is the factor significantly shaping availability of Fe (Bothe, 2015), plants occurring on soils with extremely different pH values seem to have efficient adaptations regulating acquisition of this element or wide tolerance to sub- and supraoptimal Fe availability.

Distribution of plant species in wide range of conditions as well as their ability to adjust reactions to changing environment result from evolutionary conserved adaptations (Schulze et al., 2019). Even closely related plant species can show both extremely different (vicarious plant species; Bothe, 2015) or exceptionally similar requirements for habitat (congeneric plant species; Silvertown & Wilkin, 1983). Although congeneric species (including some species of *Centaurea*) occupy the same geographical areas and co-occur in plant communities, their abilities to win a competition for resources differ due to their limitations of growth and reproduction adjustments (Gerlach & Rice, 2003; Eckhart et al., 2017) partially resulting from differences in their ability to satisfy nutritional demands. For example, such a situation is hypothetically possible for congeneric plant species co-occurring in extremely contrasting communities, namely pioneer grasslands settled on acidic and dry soils (*Koelerio-Corynephoretea* Klika in Klika et Novák 1941 class) and xerothermic grasslands established on alkaline substratum (*Festuco-Brometea* Br.-Bl. et Tüxen ex Soó 1947 class). However, it is not clear how species characterized with very wide tolerance to soil pH are able to persist and reproduce under such different conditions.

Comparisons of the soil requirements of plant species can give a valuable information about crucial determinants limiting their occurrence and co-occurrence (Soliveres et al., 2014). It can be hypothesized that congeneric plant species are expected to show some differentiation in resource utilization patterns or they occupy different microhabitats within the same type of community (*e.g*., differing in soil acidity or availability of nutrients). However, studies on congeneric plant species regarding their pH-requirements are relatively rare—there is still lack of studies focusing even on common, wild species from the temperate zone. It is also not known if these species have truly similar nutritional requirements. Therefore, our intention was to draw an ecophysiological comparison between two widely-distributed and closely-related plant species, *C. scabiosa* and *C. stoebe* (Table 1), in order to estimate their habitat preferences. It is also not known if those species are chlorotic-prone and suffer from Fe limitations (both caused by Fe starvation and over-supplementation). Thus, we checked if soils differing in their characteristics (mainly acidity) as well as availability of Fe in alkaline soil, influence performance of the selected plant species. It was intended to test the following hypotheses: (1) *C. scabiosa* and *C. stoebe* have different preferences for soil type, (2) *C. stoebe* as an invasive species is better adapted to grow in contrasting soils than *C. scabiosa*, (3) availability of Fe influences functioning of the studied species. Thus, the following questions were raised: (1) Are the studied species similar in their soil requirements? (2) Is *C. stoebe* a superior species in terms of performance on contrasting soils? (3) Is Fe and element shaping growth of the studied species?

**Table 1:** Interspecific differences in reaction to soil conditions of studied species of *Centaurea* and results of two-way ANOVA showing effects of species, treatment and their interaction on the measured traits.

Parameter	Relative difference between species (Csc:Cst ratio) and significance				*F* value and significance		
	p	r	r5	r25	(S) Species^a^ df = 1	(T) Treatment^b^ df = 3	S × T df = 3
Root FW	1.57 n.s.	2.34 n.s.	1.18 n.s.	0.78 n.s.	4.98*	1.78 n.s.	2.78 n.s.
Shoot FW	0.93 n.s.	1.13 n.s.	0.82 n.s.	0.40*	4.70*	0.45 n.s.	3.24*
Root DW	1.78 n.s.	3.21*	1.63 n.s.	1.01 n.s.	16.44***	0.92 n.s.	2.32 n.s.
Shoot DW	0.91 n.s.	1.14 n.s.	0.84 n.s.	0.41 n.s.	4.17 n.s.	0.30 n.s.	2.93 n.s.
S:R FW ratio	0.53 n.s.	0.51*	0.75 n.s.	0.50 n.s.	32.35***	2.14 n.s.	1.22 n.s.
S:R DW ratio	0.44 n.s.	0.37***	0.55 n.s.	0.42*	60.51***	2.35 n.s.	1.39 n.s.
Leaf FW	1.97***	2.47***	2.02***	1.34 n.s.	132.01***	3.65*	4.78**
Leaf DW	1.74**	2.29***	1.98***	1.20 n.s.	93.78***	1.74 n.s.	5.68**
Number of leaves	0.59*	0.58 n.s.	0.56 n.s.	0.35***	66.95***	1.90 n.s.	1.50 n.s.
Leaf area	1.70**	2.04***	1.64***	1.24 n.s.	78.58***	5.10**	2.95*
LDMC	0.89 n.s.	0.92 n.s.	0.98 n.s.	0.92 n.s.	11.09**	1.45 n.s.	0.91 n.s.
SLA	0.99 n.s.	0.89 n.s.	0.82*	1.02 n.s.	6.51*	1.66 n.s.	3.14*
IDC score^c^	1.00 n.s.	1.00 n.s.	1.00 n.s.	1.00 n.s.	1.00–	1.00–	1.00–
Chlorophyll content	0.94 n.s.	0.98 n.s.	1.00 n.s.	0.95 n.s.	2.95 n.s.	0.20 n.s.	0.67 n.s.
N	0.85 n.s.	0.92 n.s.	1.00 n.s.	0.96 n.s.	5.43*	4.48**	1.48 n.s.
F_V_/F_M_	1.02 n.s.	1.00 n.s.	1.01 n.s.	1.00 n.s.	3.20 n.s.	6.60***	1.40 n.s.
F_0_	0.99 n.s.	1.02 n.s.	0.95 n.s.	0.99 n.s.	0.55 n.s.	0.25 n.s.	0.53 n.s.
F_J_	1.00 n.s.	0.96 n.s.	0.88 n.s.	0.97 n.s.	6.10*	0.55 n.s.	1.60 n.s.
F_I_	1.10 n.s.	1.02 n.s.	0.94 n.s.	0.93 n.s.	0.04 n.s.	1.28 n.s.	2.57 n.s.
F_M_	1.06 n.s.	1.02 n.s.	0.97 n.s.	0.98 n.s.	0.19 n.s.	1.51 n.s.	0.98 n.s.
F_V_	1.08 n.s.	1.02 n.s.	0.97 n.s.	0.98 n.s.	0.49 n.s.	2.36 n.s.	1.15 n.s.
S_M_	0.93 n.s.	0.92 n.s.	1.03 n.s.	0.95 n.s.	2.92 n.s.	5.79**	0.75 n.s.
Area	1.01 n.s.	0.95 n.s.	1.00 n.s.	0.94 n.s.	0.56 n.s.	7.09***	0.27 n.s.
M_0_	0.85 n.s.	0.91 n.s.	0.84*	0.98 n.s.	19.32***	5.05**	1.72 n.s.
V_I_	1.05 n.s.	1.00 n.s.	0.96 n.s.	0.92*	2.64 n.s.	3.34*	7.08***
V_J_	0.93 n.s.	0.91 n.s.	0.86*	0.97 n.s.	15.61***	4.79**	1.34 n.s.
ABS/RC	0.90*	1.00 n.s.	0.97 n.s.	1.01 n.s.	4.93*	1.93 n.s.	3.51*
TR_0_/RC	0.91*	1.00 n.s.	0.98 n.s.	1.01 n.s.	4.09*	1.05 n.s.	3.32*
ET_0_/RC	0.96 n.s.	1.07 n.s.	1.10 n.s.	1.04 n.s.	3.50 n.s.	1.69 n.s.	2.12 n.s.
DI_0_/RC	0.84*	0.99 n.s.	0.95 n.s.	1.01 n.s.	5.09*	4.43**	2.87*
ϕE_0_	1.07 n.s.	1.07 n.s.	1.13 n.s.	1.03 n.s.	14.34***	5.63**	1.05 n.s.
PI_ABS_	1.37 n.s.	1.18 n.s.	1.35 n.s.	1.05 n.s.	13.80***	6.45***	1.43 n.s.
Root Ca	1.55 n.s.	0.79 n.s.	0.74 n.s.	0.56 n.s.	4.44*	11.26***	1.68 n.s.
Root Mg	1.13 n.s.	0.61 n.s.	0.74 n.s.	0.58*	16.60***	7.26**	4.86**
Root Fe	1.10 n.s.	0.59 n.s.	0.54 n.s.	0.70 n.s.	9.82**	10.32***	1.65 n.s.
Root Mn	1.08 n.s.	0.78 n.s.	0.73 n.s.	1.27 n.s.	0.26 n.s.	5.44**	0.89 n.s.
Root Zn	1.14 n.s.	0.78 n.s.	0.66 n.s.	0.82 n.s.	8.82**	3.85*	2.82 n.s.
Root Cu	1.04 n.s.	0.72 n.s.	0.71 n.s.	0.87 n.s.	3.75 n.s.	0.56 n.s.	0.84 n.s.
Shoot Ca	2.17***	1.91***	1.96***	1.81***	173.15***	2.86 n.s.	0.37 n.s.
Shoot Mg	1.95***	1.15 n.s.	0.98 n.s.	0.89 n.s.	6.63*	21.87***	7.00**
Shoot Fe	1.88 n.s.	1.07 n.s.	1.34 n.s.	1.23 n.s.	7.39*	4.54*	0.62 n.s.
Shoot Mn	1.14 n.s.	1.39 n.s.	1.68 n.s.	1.38 n.s.	7.86**	2.09 n.s.	0.58 n.s.
Shoot Zn	3.07**	2.60*	3.28**	2.45 n.s.	62.64***	0.62 n.s.	0.59 n.s.
Shoot Cu	0.83 n.s.	1.07 n.s.	0.62 n.s.	1.13 n.s.	0.71 n.s.	0.28 n.s.	1.17 n.s.
SAP Ca	1.01 n.s.	1.07 n.s.	1.11**	1.12**	41.12***	12.06***	3.48*
SAP Mg	1.21 n.s.	1.30 n.s.	1.16 n.s.	1.19 n.s.	20.77***	7.82***	0.43 n.s.
SAP Fe	1.34 n.s.	1.49 n.s.	1.79 n.s.	1.47 n.s.	24.73***	7.36**	0.11 n.s.
SAP Mn	1.00 n.s.	1.34 n.s.	1.44 n.s.	1.10 n.s.	10.12**	18.44***	2.12 n.s.
SAP Zn	1.48**	1.76***	2.13***	1.73***	172.21***	1.30 n.s.	2.41 n.s.
SAP Cu	0.90 n.s.	1.22 n.s.	0.96 n.s.	1.13 n.s.	0.71 n.s.	1.94 n.s.	1.44 n.s.
Root Fe:Mn ratio	1.00 n.s.	0.81 n.s.	0.76 n.s.	0.60 n.s.	11.10**	2.44 n.s.	2.12 n.s.
Shoot Fe:Mn ratio	1.70 n.s.	0.81 n.s.	0.85 n.s.	0.88 n.s.	0.39 n.s.	7.27**	1.67 n.s.

**Notes:**

* *p* < 0.05.

** *p* < 0.01.

*** *p* < 0.005.

n.s. – not significant.

a *C. scabiosa* or *C. stoebe*.

b Podzol (p), Rendzina (r) or Rendzina with addition of 5 (r5) or 25 μmol Fe-HBED kg^−1^ soil (r25).

c Due to no variance of this trait, ANOVA analysis was not conducted.

Each value is a ratio of means of a given parameter measured in Csc and Cst. The values >1 detect that trait dominate in *C. scabiosa*, whereas values <1 indicate the opposite. Differences between mean values of each parameter were checked using two-way ANOVA (followed by Bonferroni’s post-hoc test; *n* = 4–16, depending on parameter; for specific information see main text of the article and figure captions).

## Materials & Methods

### Description of the studied species

*Centaurea scabiosa* L. (abbreviated as Csc) is a perennial plant. Its root is well-developed and its stem is 30 to 150 cm high, woody, upright, angular (with rough edges) and branched from the middle (Hegi, 1954). Leaves are rough on abaxial side (rarely smooth), dark-green and pinnate with lanceolate sections. Inflorescences are settled individually at the tip of the branches. Flowers are purple, sometimes pink or white; the marginal ones are greatly enlarged and radiant, only rarely absent. It occurs on meadows, dry places, roadsides, bushy and scree slopes and in light woods, sometimes on rocks in mountainous areas (Hegi, 1954). *C. scabiosa* occurs naturally throughout Europe (except most southern and northern regions) and in Asia (up to Lake Baikal in Siberia; Hegi, 1954).

*Centaurea stoebe* Tausch (syn. *C. rhenana* Borheau; abbreviated as Cst) is a biennial to perennial plant. Its root is long, thick and woody and its stem is 20 to 90 cm high, stiffly upright, angular (rough at the edges) and branched in the middle. Its leaves are gray-green to almost green; the lowest leaves are pinnate, the following ones are pinnate and lobed, and the top ones are undivided and lanceolate. Inflorescences are very numerous (up to 200), settled individually at the tip of the branches. Flowers are bluish pink to pale pink, rarely white; the marginal ones are enlarged radiant. It occurs on sunny, grassy and rocky slopes, railway embankments, roadsides, vineyard edges and ruderal sites (Hegi, 1954). *C. stoebe* occurs naturally in Western, Southern (except Iberian Peninsula) and Eastern Europe (up to Caucasus; Hegi, 1954).

According to Ellenberg (1991), both species have very similar centers of abundance. They can be found on well-lit places, on dry and extremely dry soils that are rather infertile (Table S1). According to this author the differences between the studied species are rather slight, however, *C. scabiosa* prefers slightly wetter and more fertile soils than *C. stoebe* (Table S1). The available data indicate that *C. scabiosa* and *C. stoebe* occur in both alkaline xerothermic and dry acidic grasslands, however their requirements for soil acidity are not clear (Hegi, 1954; Ellenberg, 1991; Czyżewska, 1992; Matuszkiewicz, 2001; Kącki, Czarniecka & Swacha, 2013, as well as personal observations of M. Wala and J. Kołodziejek).

### Properties of soils, experimental setup and growth conditions

The soils used in this study were as those used in our previous investigation (for full spectrum of physical-chemical properties of the used soils see Wala et al., 2020). Entic Podzol (hereafter referred to as Podzol) characterized with low pH (pH_KCl_ = 4.3), low nutrient content (0.021% N, 56 mg P kg^−1^ soil) and high content of available Fe (503 mg kg^−1^ soil). Rendzic Leptosol (hereafter referred to as Rendzina) was characteristic of higher pH (pH_KCl_ = 7.3), higher nutritional quality (0.220% N, 46 mg P kg^−1^ soil) and lesser content of available Fe (414 mg kg^−1^ soil) than Podzol.

Seeds of *C. scabiosa* and *C. stoebe* were collected at the maturity stage in 2018 from at least 30 plants from single, representative populations grown under optimal conditions in the Didactic-Experimental Garden of the Faculty of Biology and Environmental Protection (University of Lodz), central Poland (51°78′N; 19°48′E). Subsequently, the seeds were stored in paper bags for two weeks in constant laboratory conditions (21 °C; low humidity) and all damaged, discolored and malformed ones were discarded. Then they were stratified at 5 °C for 8 weeks before being used in the experiment.

The seeds of each species were mixed before the sowing for randomization. The seedlings were established from cold-stratified seeds in garden trays in Podzol or Rendzina and then randomly-selected ones from each soil were transplanted to 1.5 dm^3^ pots filled with Podzol or Rendzina, respectively. The plants were grown under field conditions (full sunlight) in the Didactic-Experimental Garden of the Faculty of Biology and Environmental Protection (University of Lodz). Climate of this area is temperate. The seasons are clearly differentiated and typical of the temperate zone. Weather conditions of the experimental period (1st April to 1st October 2019; Fig. S1) were given according to the measurements of the Institute of Meteorology and Water Management – National Research Institute (Warszawa, Poland). The average lowest temperature during experiment was 10.1 °C (April), and the average highest temperature was 22.2 °C (June). Total precipitation during the experiment was 210.4 mm with the maximum in September and the minimum in June. The plants were grown in soil with optimal moisture (maintained with water used for the preparation of the tested Fe-HBED solutions). The plants were hand-weeded when any emergence of other plants from soil seed bank was observed.

Fe-HBED chelate (N,N′-di(2-hydroxybenzyl)ethylenediamine-N,N′-diacetic acid iron(III) sodium salt; 7% Fe; PPC ADOB, Poland) was selected as Fe carrier due to its outstanding ability to deliver Fe to plants under alkaline conditions (the process simulates Fe chelation in natural conditions; López-Rayo, Hernández & Lucena, 2009). Doses used in this study were as the same as those used in our previous investigation (Wala et al., 2020), because they allow to study individual Fe requirements of a given species (Venturas et al., 2014). After acclimatization (one week), the plants grown on Rendzina were randomly grouped and exposed to 0 (r), 5 (r5) or 25 (r25) µmol Fe-HBED kg^−1^ soil (prepared using tap water). The Podzol-grown plants (p) were treated solely only with the same volume of water without addiction of Fe-HBED (as same as the plants from Rendzina without addition of Fe-HBED). The plants were supplied with selected solutions every two weeks (5 doses in total). The experiment was set up on 1st April (seed sowing) and ended on 1st October (the day of measurements and collection of plant material). All further measurements were done on the same set of plants (four plants per treatment were used; *n* = 4).

### Measurement of growth-related traits

Growth of the studied plant species was measured in order to estimate their preferences for edaphic conditions and to detect if they encounter any Fe-specific limitations on the alkaline soil. Fresh (FW) and dry weights (DW) of roots and shoots were determined by weighting, drying and re-weighting of the plant material after storage at 60 °C (c.a. 48 h). Partitioning of FW and DW between roots and shoots was estimated as a shoot:root (S:R) ratio. Leaf morphometrical analysis was performed in order to estimate resource utilization under different edaphic conditions. All measurements pertaining to leaf traits were conducted before determination of plant weight. The leaves were counted after extraction of plants from soil. Then, two (*C. scabiosa*) or four (*C. stoebe*) fully developed and representative leaves per plant were rapidly excised and weighted (FW) on an analytical balance (the number of assayed leaves per plant depended on the number of fully developed ones in both species). Subsequently, leaf area was measured using a CI-202 portable laser area meter (CID Bio-Science, USA). Then, the leaves were separately dried and re-weighted to obtain their DW. Specific leaf area (SLA) was calculated as the area per DW of a leaf blade (Garnier et al., 2001) and expressed in cm^2^ mg^−1^. Leaf dry matter content (LDMC) was calculated as the amount of leaf DW per leaf FW and expressed in mg DW g^−1^ FW. All FW and DW measurements were taken using four plants per treatment and two (*C. scabiosa*) or four (*C. stoebe*) leaves per plant.

### Chlorosis scoring and measurements of chlorophyll content

Measurements of chlorosis (both quantitative and qualitative) were conducted in order to detect soil-dependent limitations to biosynthesis and maintenance of photosynthetic pigments (signs of nutrient scarcity) and relations between these failures and the soil Fe status. The five-grade scale (Wala et al., 2020), adapted from the previous studies on Fe-dependent chlorosis (Wang et al., 2008), was used to visually estimate chlorosis (IDC score). Four plants per treatment were used for chlorosis scoring.

Chlorophyll content was determined using non-destructive fluorescence method utilizing a portable chlorophyll content meter, CCM-300 (Opti-Sciences Inc., Hudson, NH, USA) basing on findings presented by Gitelson, Buschmann & Lichtenthaler (1999). Chlorophyll content was measured according to the standard protocol and calculated as mg m^–2^. Chlorophyll content was measured using four fully developed leaves per plant and four plants per treatment.

### Measurement of chlorophyll *a* fluorescence

Functioning of photosynthetic apparatus was inspected in order to detect if it is affected (improved or worsened) by edaphic conditions or availability of Fe on the alkaline soil. The polyphasic rise in chlorophyll *a* fluorescence (OJIP) transient was inspected with handheld PAM-type fluorometer, FluorPen FP100 (Photon System Instruments, the Czech Republic) basing on the findings of Strasser, Tsimilli-Michael & Srivastava (2004). The records were gathered during optimal weather conditions (temperature > 18 °C, no cloud cover or rainfall). Prior to the data collection, the leaves were adapted in darkness for 20 min (using standard clips with shutters) in order to maximally diminish energization-dependent fluorescence. All measurements were recorded using the standard protocol of the device. Selected parameters (Table S2) were automatically calculated by the pre-programmed equations (Strasser, Tsimilli-Michael & Srivastava, 2004). The OJIP parameters were recorded on two fully developed leaves (interveinal area) per plant and four plants per treatment. In order to show relative differences between the studied variants, fingerprints of the recorded parameters were presented using means of values normalized to the values measured on the plants grown on Rendzina (Wala et al., 2020) using standard spider plot technique (Berger et al., 2007).

### Determination of elements and their partitioning

Quantification of elements and their partitioning was conducted in order to check nutritional status of plants and thus to estimate their requirements, tolerance to changing edaphic conditions and reaction to improved Fe availability. The elemental composition of roots and shoots was determined as previously described (Wala et al., 2020) using atomic absorption spectrometry (with SpectrAA 300 spectrometer; Varian Australia Pty. Ltd., Mulgrave, VIC, Australia). The content of each element was expressed in mg g^–1^ DW (Ca, Mg, Fe, Mn and Zn) or μg g^–1^ DW (Cu). Measurements were conducted on roots and shoots (leaves used to determine leaf parameters pooled with the remaining shoot material from a given plant) of four plants per treatment. In order to assess elemental allocation equilibria and to determine plant reactions to soil nutritional status, root-shoot transportation of each element was determined using shoot allocation percentage (SAP) proposed in the previous study (Wala et al., 2020). Ratio of Fe:Mn ratios in roots and shoots (a proxy of the mechanism contributing to avoidance of Fe-dependent limitations) were calculated using previously determined Fe and Mn contents.

### Statistical analysis

Experiment was designed to tetraplicate each experimental variant for each species (*n* = 4). Normality of the data was examined with Kolmogorov–Smirnov’s test and homogeneity of variances was examined with the Brown–Forsythe’s test. The differences between treatments (p, r, r5 or r25) were detected by one-way ANOVA followed by Bonferroni’s post-hoc test. Differences between variants were accepted as significant at *p* < 0.05. To determine differences between the studied species, the plants from a given experimental variant were compared using two-way ANOVA followed by Bonferroni’s post-hoc test (differences were accepted as significant at *p* < 0.05). Interspecific differences were presented as the ratios of a given trait value measured on *C. scabiosa* and *C. stoebe*. Thus, the values > 1 detect that trait dominate in *C. scabiosa*, whereas values < 1 indicate the opposite. Two-way ANOVA was conducted to detect effect of species and treatment and their interaction on the measured traits (differences were accepted as significant at *p* < 0.05). Correlation between Fe-HBED dose (r, r5 and r25 variants) and content of assayed elements was calculated separately for roots and shoots of both species using Pearson’s correlation (differences were accepted as significant at *p* < 0.05). Principal Component Analysis (PCA) based on the correlation matrices was conducted using elemental datasets (from all tested variants, separately for roots and shoots) for generation of principal components and respective values of FWs and DWs as additional variables (in order to estimate the influence of plant nutrition on growth of the studied species). All statistical analyses were conducted using Statistica™ v. 13.3 (Tibco Software Inc., Palo Alto, CA, USA).

## Results

### Effects of edaphic conditions of growth

**Figure 1:**
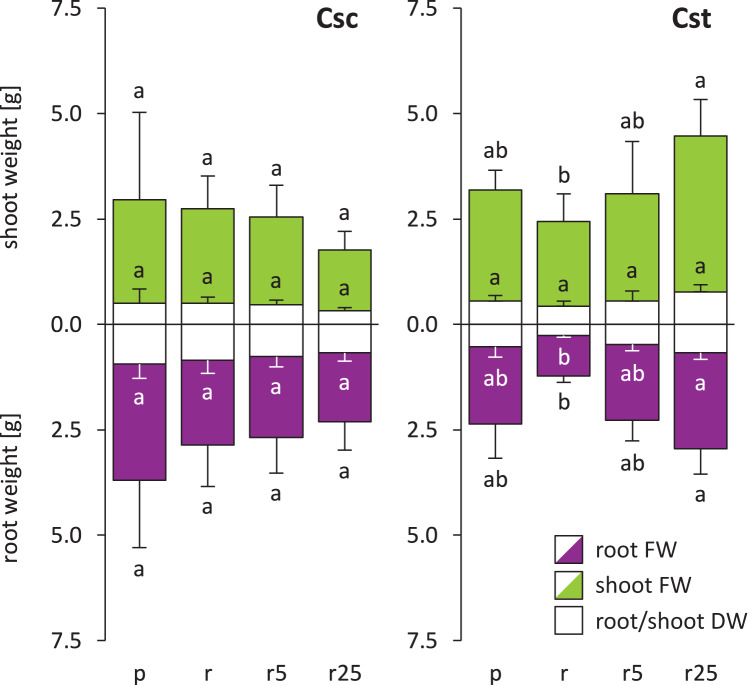
Growth of the studied species of *Centaurea* (measured as fresh weight – FW and dry weight – DW) in Podzol (p), Rendzina (r) or Rendzina with addition of 5 (r5) or 25 μmol Fe-HBED kg^−1^ soil (r25). Values (mean ± SD) with different letters are significantly different between the treatments within a given species at *p* < 0.05 (*n* = 4; ANOVA with Bonferroni’s post-hoc test). Whole bars (transparent + coloured area) – shoot/root FWs, transparent bars – shoot/root DWs.

Soil type influenced neither FW nor DW of *C. scabiosa* and *C. stoebe*, however slight preferences towards acidic soil was observed in the latter (Fig. 1). Furthermore, Fe-HBED treatments did not alter weight of *C. scabiosa*, whilst increases in FW of shoots and roots as well as in DW of roots of *C. stoebe* were observed (Fig. 1). The latter parameter was increased by c.a. 150% after application of 25 µmol Fe-HBED kg^−1^ soil, comparing to the untreated Rendzina-grown plants. Although each species differentially allocated biomass, they responded very similarly to the tested conditions, as S:R ratios of DW and FW were not altered by treatments (Fig. S2). Considering interspecific differences, *C. scabiosa* had more developed roots when grown on untreated Rendzina and definitely smaller shoots on Rendzina supplied with 25 µmol Fe-HBED kg^−1^ soil than *C. stoebe* (Table 1). Furthermore, *C. scabiosa* allocated definitely more biomass into roots, which was reflected in FW and DW S:R ratios (Table 1). Almost all abovementioned measured growth parameters (except shoot DW) depended on species, whereas treatment and interaction of species and treatment had no significant effects (except shoot FW) (Table 1).

**Figure 2:**
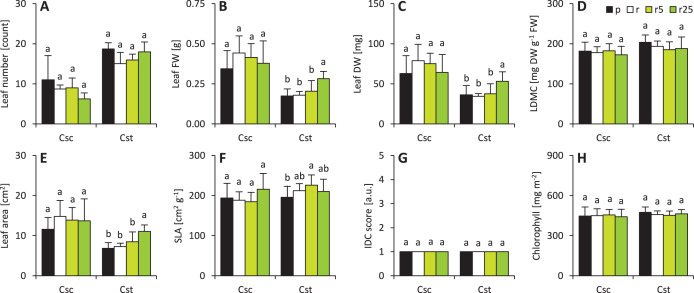
Characteristics of the leaves (A–number of leaves, B–leaf fresh weight–FW, C–leaf dry weight–DW, D–leaf dry matter content–LDMC, E–leaf area, F–specific leaf area–SLA, G–grade of iron dependent chlorosis–IDC score, H–chlorophyll). The IDC scores are as follows: 1–no visual signs of chlorosis (green leaf blades), 2–slight yellowing of leaves, 3–evident interveinal chlorosis (green veins and yellow interveinal areas), 4–interveinal chlorosis and slightly developing necrosis, 5–severe chlorosis (yellow veins and interveinal areas) and necrosis. Values (mean ± SD) with different letters for each parameter are significantly different between the treatments within a given species at *p* < 0.05 (*n* = 8 for Csc and *n* = 16 for Cst for leaf number, FW, DW, LDMC, area and SLA; *n* = 4 for IDC score and *n* = 16 for chlorophyll content; ANOVA with Bonferroni’s post-hoc test).

Soil type and Fe-HBED treatments did not affect leaf-associated traits in *C. scabiosa* (Fig. 2). On the other hand, 25 µmol Fe-HBED kg^−1^ soil increased leaf FW and DW in *C. stoebe* (Fig. 2). Furthermore, the greater the dose of Fe-HBED, the greater the leaf area of *C. stoebe* was observed (Fig. 2). Interestingly, treatment with 5 µmol Fe-HBED kg^−1^ soil caused the highest values of SLA in this species (Fig. 2). Moreover, the leaves of *C. scabiosa* were characterized with greater weight and area at the cost of their number, when compared to *C. stoebe* (Table 1). All measured leaf-associated traits were affected by species, whilst the treatment affected only leaf FW and area (Table 1). Several traits (leaf FW and DW, area, SLA) depended on the interaction between the studied factors (Table 1).

### Resistance to Fe-dependent chlorosis and chlorophyll *a* fluorescence

Both species performed without any visual signs of chlorosis which was also reflected in qualitative IDC score and quantitative measurements of chlorophyll content (Fig. 2). Interspecific differences were not observed for these parameters (Table 1). It was reflected in two-way ANOVA analysis (Table 1).

**Figure 3:**
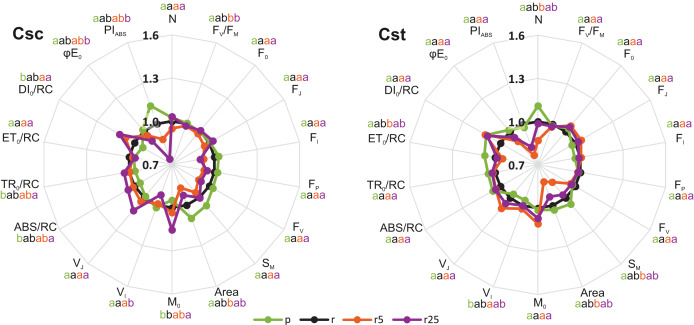
Relative changes of chlorophyll *a* fluorescence parameters (OJIP test) in leaves of the studied species of *Centaurea* grown on Podzol (p), Rendzina (r) or Rendzina with addition of 5 (r5) or 25 μmol Fe-HBED kg^−1^ soil (r25). Relative values (means) were calculated as ratios between mean values measured on plants subjected to a given treatment and mean values measured on Rendzina-grown plants. Values with different letters are significantly different between the treatments within a given species at *p* < 0.05 (*n* = 8; ANOVA with Bonferroni’s post-hoc test). The earlier letter indicates a significantly higher value of the parameter. The color of letter-based statistical indicators refers to the respective experimental variant as indicated in the legend. See Table S2 for definitions of plotted parameters.

Significant, treatment-dependent changes in OJIP parameters were only rarely observed. Podzol-grown *C. scabiosa* and *C. stoebe* showed similar performance of photosynthetic apparatus compared to the respective plants grown on Rendzina (Fig. 3). Addition of 5 µmol Fe-HBED kg^−1^ soil to Rendzina did not cause any changes in both studied species while that of 25 µmol Fe-HBED kg^−1^ soil affected only *C. scabiosa* (higher values of M_0_ and lower of V_I_) (Fig. 3) as compared to the untreated Rendzina. Most pronounced differences between the species were observed when they were grown on Podzol (ABS/RC, TR_0_/RC, DI_0_/RC, PI_ABS_) (Table 1). Furthermore, lower values of M_0_ and V_J_ were observed in Rendzina-grown *C. scabiosa* plants (supplied with 5 μmol Fe-HBED kg^−1^ soil) than in respective *C. stoebe* ones (Table 1). According to two-way ANOVA analysis, species significantly affected 9 out of 18 OJIP parameters, whereas treatment 10 out of 18 (Table 1). Only 4 out of 18 measured OJIP traits were affected by interaction between the studied factors (Table 1).

### Elemental composition, allocation of elements, Fe:Mn ratios, influence of Fe-HBED on elemental composition and relation between nutritional status and growth

**Figure 4:**
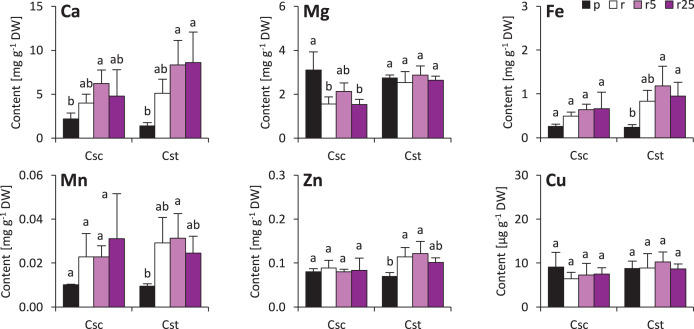
Root mineral composition (contents of calcium – Ca, magnesium–Mg, iron–Fe, manganese–Mn, zinc–Zn and copper–Cu) of the studied species of *Centaurea* grown in Podzol (p), Rendzina (r) or Rendzina with addition of 5 (r5) or 25 μmol Fe-HBED kg. Values (mean ± SD) with different letters for each parameter are significantly different between the treatments within a given species at *p* < 0.05 (*n* = 4; ANOVA with Bonferroni’s post-hoc test).

The soil type and supplementation with Fe-HBED altered the contents of several elements in roots of the studied species. That of Ca was not different considering plants grown on different soil types, however, treatment with Fe-HBED slightly increased it in both *C. scabiosa* (5 µmol Fe-HBED kg^−1^ soil) and *C. stoebe* (5 and 25 µmol Fe-HBED kg^−1^ soil) (Fig. 4). *C. scabiosa* acquired more Mg when grown on Podzol than when grown on Rendzina, whereas contents of this element were stable in *C. stoebe* among all tested variants (Fig. 4). Surprisingly, Fe content in roots of *C. scabiosa* was not affected by soil type and Fe-HBED treatment, whereas *C. stoebe* acquired more Fe after treatment with Fe-HBED when compared to the Podzol-grown plants (Fig. 4). Mn content in roots was similar in both species (Fig. 4). The roots of Rendzina-grown *C. stoebe* plants contained more Zn than those of the Podzol-grown ones, whereas in the roots of *C. scabiosa* its content was similar among all treatments (Fig. 4). Contents of Cu were stable in both studied species (Fig. 4). Comparing C. *scabiosa* with *C. stoebe* only lower contents of Mg (Rendzina-grown plants treated with the highest dose of Fe-HBED) was observed in the former species. (Table 1). Elemental composition of roots depended on species (except Mn and Cu) and treatment (except Cu) (Table 1). Interaction of these factors had effect only on Mg content (Table 1).

**Figure 5:**
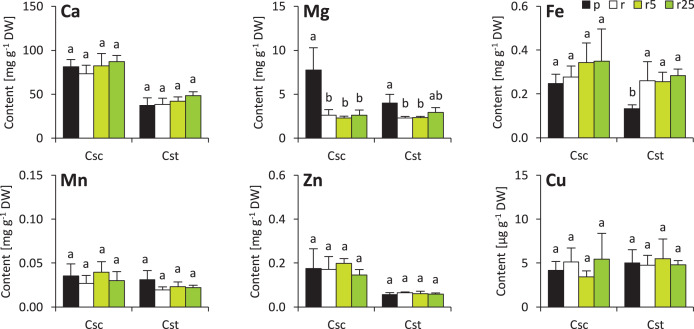
Shoot mineral composition (contents of calcium – Ca, magnesium–Mg, iron–Fe, manganese–Mn, zinc–Zn and copper–Cu) of the studied species of *Centaurea* grown in Podzol (p), Rendzina (r) or Rendzina with addition of 5 (r5) or 25 μmol Fe-HBED k. Values (mean ± SD) with different letters for each parameter are significantly different between the treatments within a given species at *p* < 0.05 (*n* = 4; ANOVA with Bonferroni’s post-hoc test).

Minor changes were observed also in shoots of the studied species. Soil type and treatment with Fe-HBED did not influence contents of Ca, Mn, Zn and Cu in the shoots (Fig. 5). On the other hand, Fe content was greater in all Rendzina-grown *C. stoebe* plants than in Podzol-grown ones (treated as well as untreated with Fe-HBED; Fig. 5). Furthermore, both species showed higher Mg contents when grown on Podzol than on Rendzina (Fig. 5). It is worth noting that the shoots of *C. scabiosa* contained c.a. 100% more Ca and c.a. 150–200% more Zn than those of *C. stoebe*, whereas the contents of the other assayed elements were similar (except Mg in Podzol-grown plants; Table 1). Shoot contents of almost all elements (except Cu) affected by species, whilst only Mg and Fe depended on treatment (Table 1). Interactions between the studied factors were showed to influence only the content of Mg (Table 1).

**Figure 6:**
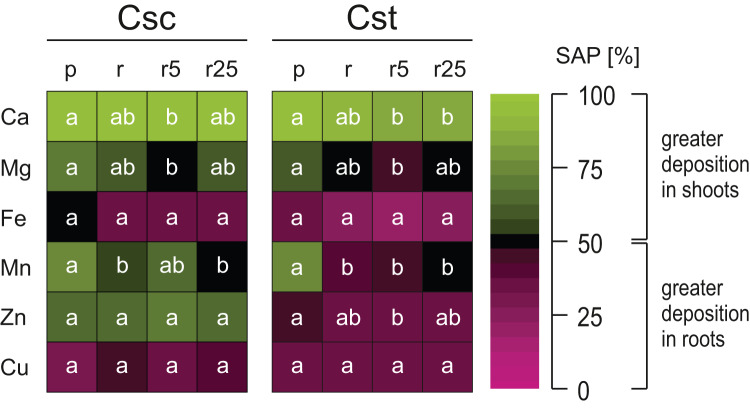
Heatmap of element partitioning measured as shoot allocation percentage (SAP) in the studied species of *Centaurea* grown on Podzol (p), Rendzina (r) or Rendzina with addition of 5 (r5) or 25 μmol Fe-HBED kg^−1^ soil (r25). Values (means) with different letters for each parameter are significantly different between the treatments within a given species at *p* < 0.05 (*n* = 4; ANOVA with Bonferroni’s post-hoc test). The earlier letter indicates significantly higher value of parameter.

Both studied species showed similar pattern of elemental allocation on the root:shoot axis, with important exceptions. They both allocated Ca in greater amount into shoots and Fe and Cu into roots (Fig. 6). However, it is worth noting that the plants grown on Rendzina supplied with Fe-HBED showed lesser Ca SAP values than those grown on Podzol (Fig. 6). Furthermore, in both species more Mg and Mn was allocated into shoots when the plants were grown on the acidic substratum than when grown on the alkaline soil (Fig. 6). Interestingly, only the allocation pattern of Zn contrasted the studied species, as *C. scabiosa* allocated this element mostly into shoots, in *C. stoebe* Zn remained in roots (Fig. 6).

Considering interspecific differences, *C. scabiosa* showed higher values of SAP than *C. stoebe*, including such elements as Ca, and Zn (Table 1). SAP values depended on species (excluding Cu) and treatment (excluding Zn and Cu) (Table 1). Only allocation of Ca was affected by interaction between the studied factors (Table 1).

**Figure 7:**
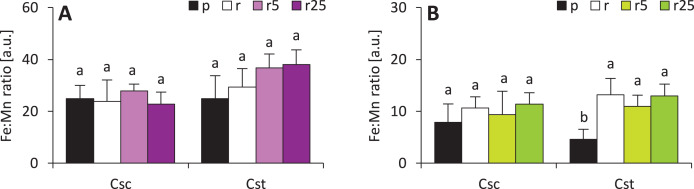
Iron to manganese ratio (Fe:Mn ratio ) in roots (A) and shoots (B) of the studied species of *Centaurea* grown on Podzol (p), Rendzina (r) or Rendzina with addition of 5 (r5) or 25 μmol Fe-HBED kg^−1^ soil (r25). Values (mean ± SD) with different letters for each parameter are significantly different between the treatments within a given species at *p* < 0.05 (*n* = 4; ANOVA with Bonferroni’s post-hoc test).

Calculated Fe:Mn ratios (known to be reflection of plant reaction to iron deficiency) showed that Fe-HBED did not affect this parameter in either species (Fig. 7). Fe:Mn ratios in roots of the studied species were statically undifferentiated, however, slightly increasing trend in *C. stoebe* was observed (Fig. 7). The only significant difference was observed between shoots of Podzol- and Rendzina-grown *C. stoebe* plants (Fig. 7). Root and shoot Fe:Mn ratios in *C. scabiosa* and *C. stoebe* plants were not statistically different (Table 1). Root Fe:Mn ratio depended only on species, whereas shoot Fe:Mn ratio only on treatment (Table 1).

**Figure 8:**
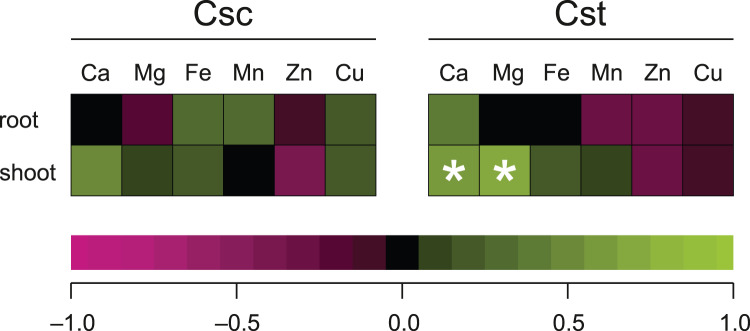
Spearman’s rank correlation between dose of chelated Fe (0, 5 and 25 μmol Fe-HBED kg^−1^ soil) and content of the analysed elements in roots and shoots of the studied species of *Centaurea* grown on Rendzina. *p < 0.05.

Significant correlation between the dose of Fe-HBED and the content of a given element was observed only in the case of Ca (R = 0.635) and Mg (R = 0.704) in shoots of *C. stoebe*, whilst all other calculated correlations were not significant (Fig. 8).

**Figure 9:**
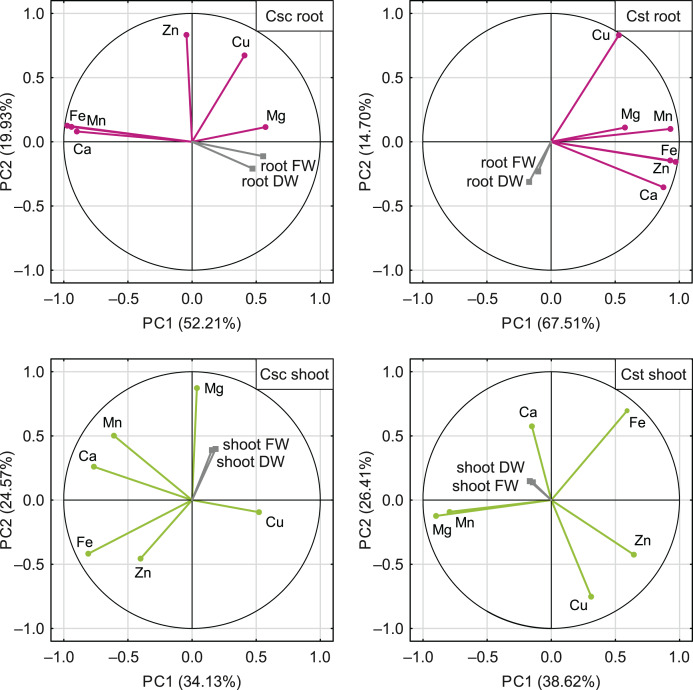
Correlation matrix of content of the analysed elements (Ca, Mg, Fe, Mn, Zn and Cu) and growth traits (root and shoot FW/DW) of the studied species of *Centaurea* plotted on two first principal components (PC) from Principal Component Analysis (PCA). Each point represents a variable, and each vector represents correlation between a given variable and PCs. Color-marked traits were used for generation of PC1 and PC2 (active variables) and grey-marked traits were plotted as additional variables. Percentages presented in parentheses indicate the explained variance. For details pertaining to the loadings of PC1, PC2 and PC3, see Table S3.

The PCA based on the data from elemental analysis showed that the first two principal components accounted for 72.14% (*C. scabiosa*) and 82.21% (*C. stoebe*) of the total variance in roots and 58.70% (*C. scabiosa*) and 65.03% (*C. stoebe*) of the total variance in shoots (Fig. 9). Considering loadings, Fe and Mn accounted the most for the first principal component in roots and Fe and Ca in shoots of *C. scabiosa* (Table S3). In the case of *C. stoebe*, Fe, Mn and Zn accounted the most for the first principal components in roots, whilst Mg and Mn in shoots (Table S3). Second principal components was associated with Zn (roots) and Mg (shoots) in *C. scabiosa*, whilst Cu loaded the most for second principal component in *C. stoebe* (Table S3). Considering third principal component, Mg (roots of both studied species) and Zn (shoots of both studied species) loaded the most (Table S3). The root and shoot FWs and DWs were positively correlated with the first principal component in *C. scabiosa*, whilst negatively in *C. stoebe* (Fig. 9). The values of root FWs and DWs in both species were negatively correlated with the second principal component, whilst the opposite was observed in the case of shoot FWs and DWs (Fig. 9).

## Discussion

Many terrestrial plant species suffer from Fe-dependent limitations (Grime & Hutchinson, 1967; Sanz, Cavero & Abadía, 1992; Tagliavini & Rombolà, 2001), however it seems not to be the case for the studied species of *Centaurea*. Development of Fe-dependent chlorosis is the best-known effect of severe Fe starvation (Lucena & Hernandez-Apaolaza, 2017). The previous study using the same experimental setup revealed that some calcicole plant species from xerothermic grasslands (*e.g. Aster amellus* L, *Betonica officinalis* L. and *Prunella grandiflora* (L.) Scholler.; Wala et al., 2020) suffered from chlorosis due to Fe-dependent limitations. Visual signs of Fe deficiency were not observed in *C. scabiosa* and *C. stoebe* in the current study, which generally suggests that soil acidity and availability of Fe are not factors strongly limiting growth and development of these species (question 1, 2 and 3). To the best of our knowledge, this is the first experimental study investigating soil preferences of those species as well as in general, plant congeners with wide ecological amplitude in terms of soil pH.

The results indicated that there were no major obstacles for establishment of *C. scabiosa* and *C. stoebe* in totally contrasting soils, as there were no significant differences pertaining to growth between the plants grown on acidic Podzol and slightly alkaline Rendzina (question 1 and 2). Similar behavior can be observed in several other dicotyledonous plants (*e.g. Dianthus carthusianorum* L, *Euphorbia cyparissias* L. and *Hypericum perforatum* L.; Stahevitch, Crompton & Wojtas, 1988; Krawczyk, Domagała-Świątkiewicz & Lis-Krzyścin, 2017) that are able to persist in extremely different communities, namely acidic dry grasslands and alkaline xerothermic grasslands. It is also known that tetraploid, polycarpic *C. stoebe* subsp. *micranthos* became an invasive species. Although adaptive differences between diploids (studied *C. stoebe*) and tetraploids (*C. stoebe* subsp. *micranthos*) are not known, it can be hypothesized that the presented results partially explain why *C. stoebe* (especially *C. stoebe* subsp. *micranthos*) colonizes new geographical areas very efficiently (Akin-Fajiye & Gurevitch, 2018), taking also into consideration the fact that polyploids are better adapted to a wide spectrum of environmental conditions (Te Beest et al., 2012). It is in agreement with the studies showing that wide tolerance to edaphic conditions allows invasive behavior of other plant species (Sanderson, Day & Antunes, 2015; Kołodziejek, 2019). Considering growth of the studied species, is not fully clear why *C. scabiosa* does not show invasive behavior. This plant is known to be a ‘stress-tolerant’ subordinate species from alkaline grasslands (Moora, Öpik & Zobel, 2004). It is probable that other characteristics, *e.g*., ability to cope with environmental stressors, pollination ecology, germination ecology and biotic interactions hinder efficient propagation of this species. Furthermore, considering reaction to soil type, interspecies differences in growth (*e.g*., weight allocation pattern and leaf characteristics) are likely to result from physiognomy of the studied species, but not from their individual reaction to substratum (alkaline Rendzina *vs* acidic Podzol). To support this finding, it can be mentioned that the studied plant species showed different weight allocation between roots and shoots, but the general trend in a given species of S:R ratio remained unaffected. Although it is still an element of scientific debate, some studies (*e.g*., Funk, 2013) suggested that high allocation of biomass into shoots was typical of invasive species (which is in agreement with the presented study considering this trait in *C. stoebe*, but not in *C. scabiosa*).

Another trait that is often assessed in studies on ability to cope with stress, competitiveness and invasiveness of plant species is SLA, as more invasive plants and species with greater amplitude of tolerance to edaphic conditions are often characterized with higher values of this parameter (Baruch & Goldstein, 1999). Although differences between the studied species were lesser than one might expect, SLA values were significantly higher in *C. stoebe* than in *C. scabiosa* in one case (r5), which is in agreement with common beliefs about the connection between SLA and invasiveness. Moreover, this parameter is sometimes described as a trait connected with assimilation rate (Storkey, 2005), however, considering OJIP parameters measured in this investigation (*e.g*., F_V_/F_M_ and PI_ABS_), it is not very probable in the case of the studied species. In general, the numbers of leaves in rosettes of both studied species were within ranges reported before (Moora, Öpik & Zobel, 2004; Henery et al., 2010). Greater number of leaves in *C. stoebe* than in *C. scabiosa* can be beneficial for the former species under the conditions provoking accelerated aging of leaves or their destruction due to environmental stress under harsh conditions of inland sand dunes (*e.g*. due to sandblasting caused by wind; Maun, 1994). In the case of *C. stoebe*, it can be also interpreted as a long-term investment in higher competitiveness (Moora, Öpik & Zobel, 2004). However, none of the tested variants influenced leaf traits in C. *scabiosa*. It is a relatively rare situation, as contrasting edaphic conditions and thus nutrient availability (including Fe) were showed to affect leaf lamina morphometric parameters (McDonald et al., 2003; Masuda et al., 2018; Wala et al., 2020). This supports the conception that *C. scabiosa* is in general species a with wide ecological amplitude (with regard to soil preferences), at least considering theoretical niche optima. Rendzina supplemented with 25 µmol kg^−1^ soil Fe-HBED promoted increase in leaf weight and area in *C. stoebe*, even comparing to the Podzol-grown plants. This suggests that growth and development of this plant on calcareous grasslands is at least partially dependent on interactions with neighboring plants and soil microbiota, both regulating availability of Fe (Colombo et al., 2014).

Looking through the prism of stable co-existence of congeneric species, at least minimal differences (*e.g*., related to individual nutrition requirements) between them must occur (Silvertown & Wilkin, 1983). The major difference between the studied species consisted in their reactions to increasing availability of Fe on the alkaline soil. Application of Fe-HBED into Rendzina was beneficial only for *C. stoebe*, which was reflected in growth-related parameters. It suggests that availability of Fe can be a limiting factor for this species growing on an alkaline soil, because promotion of growth by addition of a given element is known to be a sign of a specific limitation. It suggests that the edaphic optimum of *C. stoebe* is associated with acidic soils (with high availability of Fe) or soils on which geochemical and biological processes promote solubilization of Fe-containing compounds. However, application of the chelate did not trigger any significant changes in the elemental status in shoots and roots of this species (including Fe content). It is possible that the improved growth after application of Fe-HBED resulted from altered availability and/or acquisition of other, non-assayed elements (*e.g*., macro- and micronutrients or ballast elements; Baxter et al., 2008), which optimize nutritional status of plants. It is also possible, that increasing availability of Fe unburdened secondary metabolism due to Fe-availability-dependent stimuli (as there is a negative feedback loop between iron availability and phytosiderophore release; Ma et al., 2003; Chen, Wang & Yeh, 2017). It is known that *C. stoebe* exudates (±)-catechin into rhizosphere (Blair et al., 2006). This flavonoid-type polyphenolic compound is classified as phytosiderophore due to its catechol moiety covering chelation of various metals, including Fe (Chobot & Hadacek, 2010). If exudation of (±)-catechin substantially contributes to Fe scavenging in *C. stoebe* under physiological conditions, carbon skeletons saved from siderophore biosynthesis pathway (due to increased availability of Fe) are likely to be redirected into other metabolic processes, including also those associated with plant growth *per se*. This explanation supports the conception that the primary role of (±)-catechin consists in metal chelation (Blair et al., 2006; Chobot et al., 2009). Unfortunately, nothing is known about root exudation patterns of *C. scabiosa*. However, the presented data clearly showed that Rendzina-grown *C. scabiosa* had higher contents of Fe, Mn and Zn in roots than the Podzol-grown plants, which suggests that induction of phytosiderophore biosynthesis is triggered by alkaline substratum in this species as well. Similar mode of action was reported also in a wide spectrum of calcicole and calcifuge plant species (Ström, Olsson & Tyler, 1994). On alkaline soil phytosiderophore-caused nutritional improvements are more probable than those associated with enhanced rhizospheral acidification, due to very high buffering capacity of soil carbonate buffer, making rhizospheral acidification tough (Grillet & Schmidt, 2017). However, it is worth noting that plentiful exudates are not produced by plants when they encounter outstanding availability of divalent ions (*e.g*., on acidic soils).

Acquisition of Fe and Mn on acidic soil is an interesting aspect of microelemental balance in the studied species. The contents of these elements are definitely lower than in the plants grown on Rendzina and even lower than in some remarkably calcicole species grown on the same acidic and sandy soil (Wala et al., 2020). It indicates that minimal nutritional demands of the studied species of *Centaurea* are low. It also suggests that acquisition of several divalent ions from alkaline soils is phytosiderophore- and/or pH-dependent in these species. Furthermore, these plants were able to satisfy their Fe demands on alkaline soil, which coincided with increasing content of Mn in their tissues. It resulted in more or less stable Fe:Mn ratio, which implies that the studied species are adapted to tolerate increased Mn uptake as a side effect of Fe scavenging. It is worth noting that low Fe:Mn ratio is known to promote chlorosis (Gama et al., 2016; Zamboni et al., 2017; Wala et al., 2020). However, the studied species of *Centaurea* are able to operate without signs of chlorosis when this parameter is even lower than in chlorosis-resistant calcicole plant species from xerothermic grasslands, *e.g*., *Salvia verticillata* L. or *Veronica teucrium* L. (Wala et al., 2020). Considering the molecular mechanism of this adaptation, acquisition and vacuolar storage of excessive Mn (allowing functioning of Fe scavenging system) is the most plausible one, as this mechanism is known to support optimal functioning of Fe uptake (Eroglu et al., 2016).

Testing of chlorophyll *a* fluorescence was showed by numerous studies to be a tool allowing diagnosis of physiological state of a plant (Kalaji et al., 2016; Stirbet et al., 2018). For example, several investigations showed that some of OJIP parameters could be used for discrimination between plants tolerant and intolerant to deficiency and/or excess of Fe (Luna et al., 2018, Wala et al., 2020). Both studied species did not show any symptoms of Fe- and/or CaCO_3_-dependent limitations concerning photosynthetic apparatus functioning that are known from other plants species, *e.g*., *Vitis vinifera* L. (Shahsavandi et al., 2020). On the other hand, slightly decreased values of PI_ABS_, ϕ_E0_ and F_V_/F_M_ as well as increased values of DI_0_/RC, ABS/RC and M_0_ in *C. scabiosa* plants after application of 25 µmol Fe-HBED kg^−1^ soil suggest that this species might not tolerate high availability of Fe in alkaline soil. Similar mode of action was reported in sweet potato (*Ipomoea batatas* L.) plants oversupplied with Fe (Adamski et al., 2011). It probably has some ecological implications for the studied species of *Centaurea*. Several studies showed that graminoid plant species exudate large quantities of Fe chelators, which modulate availability of this element for other plant species, including dicotyledons (Dai et al., 2019). Thus, it is very likely, that grasses being an important element of xerothermic grasslands (*e.g*., *Brachypodium pinnatum* L, whose dominance has adverse effects on plant diversity; Bobbink & Willems, 1987) negatively affect *C. scabiosa*, making it a subordinate species due to solubilization of Fe-containing soil compounds. In contrast, such a situation could be beneficial (or at least neutral) for *C. stoebe*.

Comparing other aspects of elemental composition of the studied species, two major differences between them can be observed – *C. scabiosa* acquired greater amounts of Ca and Zn than *C. stoebe* (although relations between the tested variants within a given species were similar). *C. scabiosa* allocated Ca and Zn into shoots and roots, respectively. It is in agreement with the studies showing that *C. scabiosa* stores excess of Ca in leaf trichomes (probably in an oxalated form; De Silva, Hetherington & Mansfield, 1996). Besides natural and seminatural types of communities considered in this article (alkaline xerothermic and acidic dry grasslands), anthropogenic calamine soils rich in Zn were showed as substrata supporting growth of *C. scabiosa* and *C. stoebe* (Skubała, 2011; Pająk et al., 2018). Considering this information and the data presented in this study (including acquisition and allocation of Zn on the root-shoot axis), it can be proposed that the studied species have different strategies allowing their persistence on alkaline soils with at least average content of Zn; *C. scabiosa* can be treated as a shoot Zn accumulator (due to allocation of this element into shoot), whereas *C. stoebe* avoids acquisition of this element and its translocation to shoots. Similar differentiation (Brown et al., 1995) was showed between a Zn accumulator (*Thlaspi caerulescens* J. Presl & C. Presl) and a plant tolerant to Zn (*Silene vulgaris* (Moench) Garcke). The difference pertaining to Zn acquisition explains also why *C. scabiosa* can be found predominantly on more or less basic soils (*e.g*., Rendzinas), even those rich in Zn (*e.g*., post-mining ones; Pająk et al., 2018).

Comparing the Rendzina- and Podzol-grown plants, accumulation of Mg in aboveground organs was observed in the latter ones. Lesser availability of Mg in alkaline soils is known to be a result of soil pH (Senbayram et al., 2015) and high availability of Ca (Guo et al., 2016). However, similar limitations were observed on remarkably acidic soils due to H^+^ enrichment of soil solution (Senbayram et al., 2015). The presented results indicate that low soil pH, and thus high H^+^ and Al^3+^ concentrations, were not an obstacle for Mg scavenging in the studied species of *Centaurea*. Furthermore, even the low content of Mg recorded in the Rendzina-grown plants did not trigger any detectable decrease in chlorophyll content nor severe malfunctions of photosynthetic apparatus. Interestingly, negative coincidence between Fe and Mg contents, which was observed in the presented study, was previously shown in some species, (*e.g*., *Ulmus laevis* Pall. and *U. minor* Mill.; Venturas et al., 2014). Although this phenomenon was described in the past (Fageria, 2001), it is not known if Mg and Fe antagonism results from cause-effect relation or it just occurs due to Ca enrichment of soil. Additionally, accumulation of Mg may have a protective role when Al availability in soil is relatively high (Bose, Babourina & Rengel, 2011), which suggests additional adaptation allowing those species persistence on remarkably acidic soils, *e.g*., Podzols (where the pool of available Al is high, especially at pH lower than five; Abedi, Bartelheimer & Poschlod, 2013).

## Conclusions

In conclusion, both species are able to survive and develop under extremely different edaphic conditions. Both of them are well adapted xeric plants. However, the presented investigation indicated that ecophysiological optimum of *C. scabiosa* was shifted to alkaline soils (due to its reaction to Fe-HBED application into Rendzina as well as acquisition of Ca and Zn), whilst soil preference of *C. stoebe* was tilted towards acidic soils (or other soils with increased availability of Fe; most notably due to its reaction to Fe-HBED and physiognomy). *C. scabiosa* and *C. stoebe* are species invulnerable to severe Fe-dependent malnutrition. Even more, they have wide tolerance to nutritional soil status, as no serious symptoms of limitations were observed in this study. It can be proposed that high tolerance to edaphic conditions and low requirements of those species contribute to their increasing geographical range and/or high ability to persist in tough habitats. Their physiological adaptations allow them to colonize new areas, even on sites where patchy pattern of soil makes spread of other species tough. It is also worth noting that both species are able to develop without any signs of Fe-dependent chlorosis when grown in separation from other species. It means that the tested species do not necessarily require the common good of mobilized Fe (*e.g*., from activity of other plants) to survive. This gives them advantage over other species during establishment on vegetation gaps and loose vegetation sites (*e.g*., psammophilous grasslands).

## Supplemental Information

10.7717/peerj.12417/supp-1 Supplemental Information 1 Mean temperature and precipitation in Łódź city during experiment (April‑September 2019).

10.7717/peerj.12417/supp-2 Supplemental Information 2 Fresh weight (FW; A) and dry weight (DW; B) partitioning calculated asshoot:root (S:R) ratio of the studied species of *Centaurea* grown in Podzol (p), Rendzina (r) orRendzina with addition of 5 (r5) or 25 μmol Fe-HBED kg^−1^ soil (r25). Values (mean ± SD) withdifferent letters for each parameter are significantly different between the treatments at *p* < 0.05 (*n* = 4; ANOVA with Bonferroni’s post-hoc test).

10.7717/peerj.12417/supp-3 Supplemental Information 3 Ecological indicator values describing realized niche optima of the studied species of *Centaurea*. Ordinal scale (1–9) of Ellenberg’s Indicator Values follows Ellenberg (1991). L,light requirements ranging from 7 to 8, where 7 indicates semi-lit conditions (c.a. 30% of relative illumination) and 8 indicates light conditions (c.a. 40% of relative illumination); T, temperature requirements of 7 indicating species preferring warm conditions (characteristic of North European Plain); K, continentiality requirements ranging 3 to 5, where 3 indicates atlantic/subatlantic conditions and 5 indicates subatlantic/subcontinental conditions; F, soil moisture requirements ranging 2 to 3, where 2 indicates dry and extremely dry soils and 3 indicates dry soils; R, soil pH requirements of 8 indicating average basic soils originating from limestones; N, nitrogen availability requirements ranging 3–4, where 3 indicates slightly fertile soils and 4 denotes slightly and intermediately fertile soils; 0 – indifferent behavior, wide amplitude or unequal behavior indifferent areas.

10.7717/peerj.12417/supp-4 Supplemental Information 4 Parameters derived from the OJIP transient used in this study, formulas of their calculation and definitions. List of studied parameters follows Wala et al., 2020.

10.7717/peerj.12417/supp-5 Supplemental Information 5 Loading values of Principal Component Analysis (PCA) for the three first components (PC1, PC2 and PC3).

10.7717/peerj.12417/supp-6 Supplemental Information 6 Raw data of measured traits. Growth, OJIP, chlorosis and elemental analyses.

## Additional Information and Declarations

### Data Availability

The following information was supplied regarding data availability: This raw data from all experiments including measurements of growth, chlorosis, chlorophyll fluorescence and elemental composition are available in the Supplemental File.
