# Supplementary material for: Reactions of two xeric-congeneric species of Centaurea (Asteraceae) to soils with different pH values and iron availability
Source: PeerJ. 2021 Nov 10;9:e12417. doi: 10.7717/peerj.12417 (PMC8590394; doi:10.7717/peerj.12417)
Supplement: Supplemental Information 1 [file peerj-09-12417-s001.docx]

**Supplemental Figure 1. Mean temperature and precipitation in Łódź city during experiment (April‑September 2019)**
