# Supplementary material for: Reactions of two xeric-congeneric species of Centaurea (Asteraceae) to soils with different pH values and iron availability
Source: PeerJ. 2021 Nov 10;9:e12417. doi: 10.7717/peerj.12417 (PMC8590394; doi:10.7717/peerj.12417)
Supplement: Supplemental Information 2 — Values (mean ± SD) withdifferent letters for each parameter are significantly different between the treatments at p < 0.05 (n = 4; ANOVA with Bonferroni’s post-hoc test). [file peerj-09-12417-s002.docx]

|  |  |
| --- | --- |

**Supplemental Figure 2. Fresh weight (FW; A) and dry weight (DW; B) partitioning calculated as shoot:root (S:R) ratio of the studied species of *Centaurea* grown in Podzol (p), Rendzina (r) or Rendzina with addition of 5 (r5) or 25 μmol Fe-HBED kg^-1^ soil (r25).** Values (mean ± SD) with different letters for each parameter are significantly different between the treatments at *p* < 0.05 (n = 4; ANOVA with Tukey’s HSD post-hoc test).
