# Supplementary material for: Reactions of two xeric-congeneric species of Centaurea (Asteraceae) to soils with different pH values and iron availability
Source: PeerJ. 2021 Nov 10;9:e12417. doi: 10.7717/peerj.12417 (PMC8590394; doi:10.7717/peerj.12417)
Supplement: Supplemental Information 3 — Ordinal scale (1–9) of Ellenberg’s Indicator Values follows Ellenberg (1991). L,light requirements ranging from 7 to 8, where 7 indicates semi-lit conditions (c.a. 30% of relative illumination) and 8 indicates light conditions (c.a. 40% of relative illumination); T, temperature requirements of 7 indicating species preferring warm conditions (characteristic of North European Plain); K, continentiality requirements ranging 3 to 5, where 3 indicates atlantic/subatlantic conditions and 5 indicates subatlantic/subcontinental conditions; F, soil moisture requirements ranging 2 to 3, where 2 indicates dry and extremely dry soils and 3 indicates dry soils; R, soil pH requirements of 8 indicating average basic soils originating from limestones; N, nitrogen availability requirements ranging 3–4, where 3 indicates slightly fertile soils and 4 denotes slightly and intermediately fertile soils; 0 – indifferent behavior, wide amplitude or unequal behavior indifferent areas. [file peerj-09-12417-s003.docx]

**Supplemental Table 1. Ecological indicator values describing realized niche optima of the studied species of *Centaurea*.** Ordinal scale (1-9) of Ellenberg’s Indicator Values follows Ellenberg (1991). L, light requirements ranging from 7 to 8, where 7 indicates semi-lit conditions (c.a. 30% of relative illumination) and 8 indicates light conditions (c.a. 40% of relative illumination); T, temperature requirements of 7 indicating species preferring warm conditions (characteristic of North European Plain); K, continentiality requirements ranging 3 to 5, where 3 indicates atlantic/subatlantic conditions and 5 indicates subatlantic/subcontinental conditions; F, soil moisture requirements ranging 2 to 3, where 2 indicates dry and extremely dry soils and 3 indicates dry soils; R, soil pH requirements of 8 indicating average basic soils originating from limestones; N, nitrogen availability requirements ranging 3–4, where 3 indicates slightly fertile soils and 4 denotes slightly and intermediately fertile soils; 0 – indifferent behavior, wide amplitude or unequal behavior in different areas.

| Name | Abbreviation | Ellenberg’s Indicator Value | | | | | |
| --- | --- | --- | --- | --- | --- | --- | --- |
|  |  | L | T | K | F | R | N |
| *Centaurea scabiosa* L. | Csc | 7 | 0 | 3 | 3 | 8 | 4 |
| *Centaurea stoebe* Tausch | Cst | 8 | 7 | 5 | 2 | 8 | 3 |
