# Supplementary material for: Reactions of two xeric-congeneric species of Centaurea (Asteraceae) to soils with different pH values and iron availability
Source: PeerJ. 2021 Nov 10;9:e12417. doi: 10.7717/peerj.12417 (PMC8590394; doi:10.7717/peerj.12417)
Supplement: Supplemental Information 5 [file peerj-09-12417-s005.docx]

**Supplemental Table 3. Loading values of Principal Component Analysis (PCA) for the three first components (PC1, PC2 and PC3).**

|  | Csc | | | | | | Cst | | | | | |
| --- | --- | --- | --- | --- | --- | --- | --- | --- | --- | --- | --- | --- |
| Variable | root | | | shoot | | | root | | | shoot | | |
|  | PC1 | PC2 | PC3 | PC! | PC2 | PC3 | PC1 | PC2 | PC3 | PC1 | PC2 | PC3 |
| Ca | 0.257246 | 0.005445 | 0.140939 | 0.286050 | 0.045773 | 0.070116 | 0.189223 | 0.141798 | 0.005283 | 0.009848 | 0.208068 | 0.031800 |
| Mg | 0.104464 | 0.010976 | 0.575275 | 0.000698 | 0.517739 | 0.085120 | 0.081836 | 0.013928 | 0.872527 | 0.347898 | 0.009619 | 0.018384 |
| Fe | 0.302531 | 0.013303 | 0.023744 | 0.320113 | 0.118556 | 0.000615 | 0.233180 | 0.027611 | 0.010010 | 0.150875 | 0.305876 | 0.059267 |
| Mn | 0.281743 | 0.011454 | 0.007787 | 0.181540 | 0.171010 | 0.120975 | 0.214145 | 0.011485 | 0.091660 | 0.270671 | 0.005278 | 0.159999 |
| Zn | 0.000594 | 0.579445 | 0.168581 | 0.078565 | 0.140862 | 0.431413 | 0.212744 | 0.023883 | 0.000472 | 0.178825 | 0.114252 | 0.169807 |
| Cu | 0.053422 | 0.379377 | 0.083670 | 0.133033 | 0.006060 | 0.291758 | 0.068872 | 0.781295 | 0.020046 | 0.041884 | 0.356907 | 0.031800 |
